# A 17-year-old male with a Small Bowel Neuroendocrine Tumor: flushing differential diagnosis

**DOI:** 10.1186/s40413-017-0161-4

**Published:** 2017-09-04

**Authors:** Maria Alejandra Forero Molina, Elizabeth Garcia, Deyanira Gonzalez-Devia, Rafael García-Duperly, Alonso Vera

**Affiliations:** 1 Faculty of Medicine, Universidad de los Andes, Bogotá, Colombia; 2 Hospital Universitario Fundación Santa Fe de Bogotá, Av 9 N° 116–20, oficina 213, Bogotá, D.C, Colombia; 3 Allergy Section, Hospital Universitario Fundación Santa Fe de Bogotá, Bogotá, Colombia; 4 Department of Internal Medicine-Endocrinology, Hospital Universitario Fundación Santa Fe de Bogotá, Bogotá, Colombia; 5 Department of Surgery, Hospital Universitario Fundación Santa Fe de Bogotá, Bogotá, Colombia

**Keywords:** Flushing, Neuroendocrine tumors, Carcinoid syndrome, Food allergies, Carcinoid tumor

## Abstract

**Background:**

Neuroendocrine tumors (NETs) are heterogeneous neoplasms that originate from cells with a secretory function. Small bowel NETs (SB-NETs) are related to serotonin hypersecretion which causes: flushing, diarrhea, abdominal pain, bronchoconstriction and heart involvement, also known as carcinoid syndrome (CS). CS can be confused with an allergic reaction and thus should be considered as a differential diagnosis in the allergy consult. We present the case of a pediatric patient initially referred under the suspicion of food allergies.

**Case presentation:**

We present the case of a 17-year-old male with evanescent non-pruriginous erythematous lesions- flushing that appeared with food consumption, associated with conjunctival injection, warmth and diaphoresis after the lesions disappeared. He denied abdominal pain, diarrhea, cough or wheezing. The 24-h urinary 5-hydroxyindoleacetic acid (5-HIAA) excretion was elevated. The CT scan showed thickening of the distal ileum and multiple lesions on both hepatic lobules and the colonoscopy revealed a tumor in the ileocecal valve. Hepatic and intestinal biopsies reported a well-differentiated NET of the ileocecal valve with hepatic metastasis. He was started on octreotide and underwent a wide hepatectomy and right hemicolectomy with improvement of symptoms.

**Conclusions:**

NETs can present as carcinoid syndrome (flushing, diarrhea, abdominal pain, wheezing), which constitutes vague symptomatology and represents a challenging diagnosis for physicians. They can be confused with an allergic reaction and the allergist should consider it as a differential diagnosis. Accurate diagnostic tests will help to diagnose NETs earlier and potentially prevent carcinoid heart disease, bowel obstruction, and improve quality of life and mortality in these patients.

## Background

NETs are heterogeneous malignant neoplasms, which originate from cells with a secretory function within the neuroendocrine system [1]. The median age of diagnosis is 66 years-old [2] and it tends to be more common in females [3–6]. NETs are uncommon in the pediatric population with an incidence around 0.995 cases per 100,000 in patients under 20 years old [3]. There is an under diagnose of this pathology related to a low index of suspicion and relatively non-specific symptoms [7]. Nonetheless, the incidence rate has increased over the last few decades [8, 9] given the increased detection of this pathology [10]. Data from the National Cancer Institute’s Surveillance, Epidemiology and End Results (SEER) shows NETs constitute a relevant cancer threat despite their low incidence rate [11]. One of the largest case series of NETs in kids [12] describes the difference between the pediatric population and adults. For instance, in children NETs develop more frequently in the appendix, with a more indolent course, as the majority does not have metastasis. The study shows evidence about the lack of standardized care as even the most common NET in kids, appendiceal [3, 6, 13], does not have proper guidelines for surgical treatment [12].

SB-NETs occur more often during the sixth and seventh decade of life [14, 15]. The annual age-adjusted incidence in the United States was 1.05 per 100,000 persons in 2012 [9]. They are extremely rare in children and up to 30% of these tumors present in multiple sites [15–17]. SB-NETs grow slowly and tend to metastasize to the mesenteric root nodes and the liver. Approximately 80% of the mortality of these tumors is due to liver failure and 16% is due to bowel obstruction [18]. The overall survival (OS) depends on many factors, such as the stage or the extent of the tumor, histology (grade), depth of the tumor invasion, gender, race and age. In general, the OS reported for 5 years is 83% [19]. Localized disease has a median survival of 14 years, while distant metastases have 5.83 years [9]. In the case of loco-regional disease the 5-year survival is 65%. In well to moderately differentiated SB-NETs the 5-year and 10-year OS rates according to the extent were: 65% and 49% for localized disease; 71% and 46% for regional metastasis; 54% and 30% for distant metastasis [20, 21]. Finally, poorly differentiated SB-NETs have a 30 to 33 months OS [9]. Taking these numbers into account, it is evident the impact this disease has on mortality and the importance of early diagnosis.

The diagnosis of SB-NETs at an early stage is often difficult because the primary tumors tend to be small and generally do not lead to symptoms [22]. Upon diagnosis, 29% of SB-NETs cases are at a localized stage, 41% are in a loco-regional stage and 30% already have metastases [2, 15]. The metastasis rate in tumors smaller than 1 cm to the lymph nodes is 12% while distant metastasis is 5%. For tumors greater than 2 cm these rates are 85 and 47%, respectively [14]. In primary SB-NETs peritoneal carcinomatosis may be present in up to 30% of the patients [23]. SB-NETs are usually diagnosed at an advanced stage and their discovery is related to the manifestation of a local complication of the tumor [24]. Maglinte et al. explored the reasons behind the delayed diagnosis of these malignancies. They found that different circumstances contributed to the diagnostic delay. For instance, if the patient failed to report the symptoms, there was less than a 2-month delay in the diagnosis; if the physicians did not order the appropriate tests, the delay was 8.2 months and if the radiologist failed to make the diagnosis there was a 12-month delay [25].

The most commonly involved type of cells in SB-NETs are the enterochromaffin (EC) cells, which are in charge of producing and storing 5-HT (serotonin). This substance is released from EC cells by different stimuli like mechanical stimulation, nutrients and chemical stimulators such as acetylcholine (Ach) [26]. In the case of SB-NETs, hypersecretion of 5-HT can cause allergy-like symptoms such as diarrhea, flushes, bronchoconstriction and heart involvement, in the form of plaque-like deposits in the heart valves [2, 22, 26]. These symptoms are known as carcinoid syndrome (CS), which is usually related to metastasis [15, 27]. In cancer registries 3–19% of patients with a SB- NET have CS, but in specialized centers the incidence has been reported as high as 71% [28–31]. CS constitutes an unusual presentation [4, 27, 32], especially in the pediatric population, as they usually have a localized disease [1, 11, 13].

To better understand CS, it is important to define flushing. It is the changes in cutaneous blood flow accompanied by reddening of the skin; it can be divided into episodic and persistent. Flushing tends to favor face, neck and upper torso, because of the relative increased volume of visible superficial cutaneous vasculature in these zones [33]. Differential diagnosis of flushing encompasses a wide spectrum of benign and malignant entities. Because of this, it is important that clinicians look for associated symptoms in order to discern the etiology. Nonetheless, the majority of conditions that present with flushing do have overlapping symptoms [33]. The flush characteristics in NETs can help to establish the location of the tumor. Midgut tumors present with a rapid cyanotic flushing, lasting for less than a minute and are often associated with a mild burning sensation. On the other hand, foregut tumors cause pruritic wheals over the entire body [33]. Again, it is important to take into account that these symptoms usually relate to the spread of the tumor.

NETs are challenging due to their unspecific symptoms [11, 34, 35]. CS components can be confused with an allergic reaction and should be considered as a differential diagnosis in the allergy consult. They are also challenging given their unpredictable behavior. After all, most of the patients with small bowel NETs tend to have distant tumor spread but with a low-grade disease, which makes precise prognostication and management quite complex [34]. We present the case and diagnostic approach of a pediatric patient initially referred to the allergy specialist under the suspicion of food allergies.

## Case presentation

A 17-year-old male was remitted to the allergist because he presented evanescent, erythematous lesions with any food consumption. He had been seen by other specialists because of this symptomatology and given the relation between the occurrence of symptoms and food ingestion there was the suspicion of food allergies. Along with these lesions he also presented conjunctival injection, warmth and diaphoresis after the lesions disappeared. He stated circumstances like standing for a long time, valsalva and strong emotions also triggered the symptoms. He denied abdominal pain, diarrhea, coughing or wheezing.

**Fig. 1:**
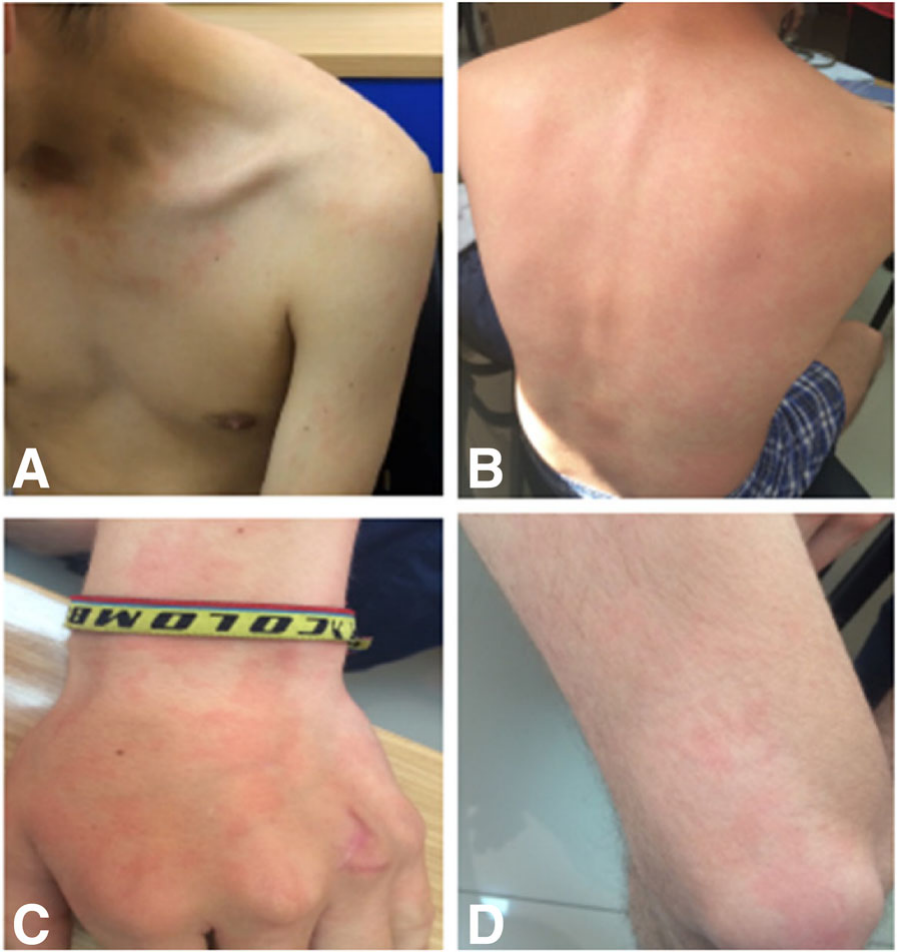
Evanescent flushing on **a** The chest, **b** The back, **c** The hand, **d** The leg

The initial physical exam was normal; no lesions were observed. While in consult we decided to trigger the symptoms. The patient was asked to eat; the first time he had coffee and a piece of chocolate cake. Nine minutes after the ingestion he presented an evanescent erythematous rash on the face, trunk and extremities (Fig. 1), which lasted 12 min and resolved spontaneously. The lesions were flat, non-pruriginous, nor painful and didn’t leave any bruising after disappearing. Given the characteristics of the lesions, the diagnosis of urticaria was discarded. In order to dismiss the suspicion of a food allergy, we asked him to drink only water and once again the rash appeared. We consequently established there was no correlation with any specific food. Based on the characteristics of the rash, we determined it corresponded to a flushing, with an atypical presentation, as it was present in lower extremities and hands.

**Fig. 2:**
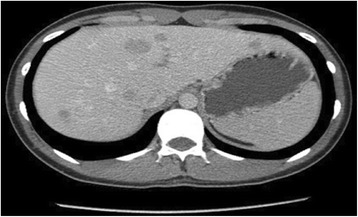
Abdominal CT-scan showing liver metastasis

**Fig. 3:**
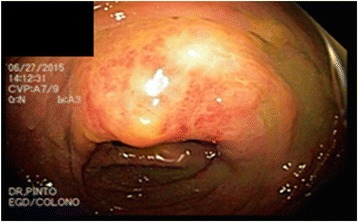
Colonoscopy with primary suspected tumor

**Fig. 4:**
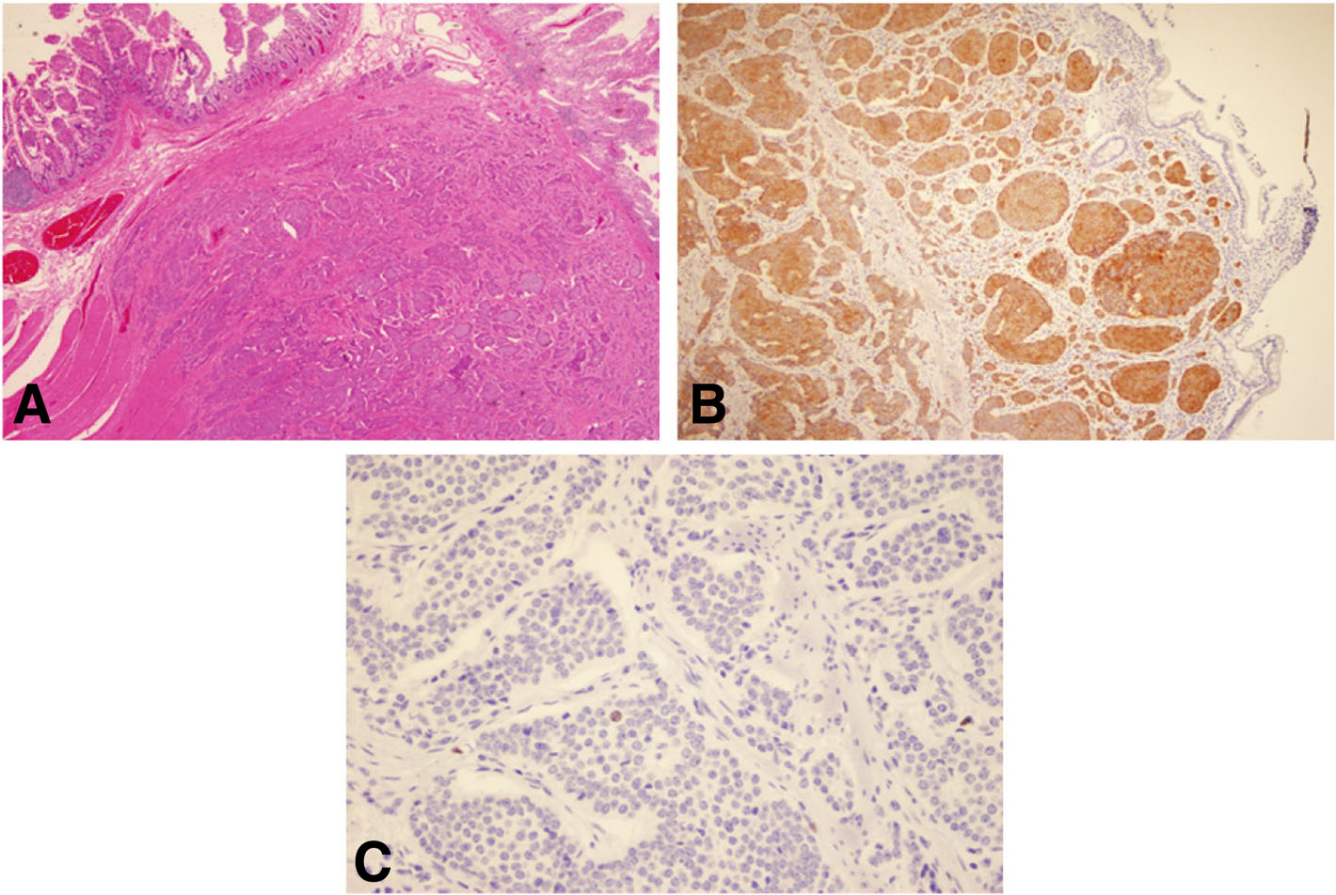
Ileocecal valve mucosa biopsy showing a grade 2, well-differentiated NET **a** Hematoxylin and eosin (H&E) staining **b** Intense reactivity to chromogranin staining **c** KI 67 staining determined a 1% proliferation cellular index

As the patient only presented flushing, we considered carcinoid syndrome an unlikely differential diagnosis, because it usually includes abdominal pain, diarrhea, wheezing or bronchoconstriction. Also, carcinoid syndrome is related to the release of serotonin in NETs, which is an improbable pathology in a teenager. Nonetheless, during his workup the 24-h urinary sample of 5-hydroxyindoleacetic acid, a metabolite of serotonin, was 42.6 mg, reference value is less than 10 mg/24 h and serum chromogranin A was normal. It was unusually high taking into account the patient, as part of the preparation for this test, was avoiding drugs and foods with high serotonin levels such as avocado, walnuts, banana, pineapple, among others [2, 36]. Therefore, the flushing observed was somehow associated to endogenous serotonin hypersecretion. There was a high suspicion of a NET and thus we ordered a CT scan. The CT scan showed thickening of the distal ileum and multiple lesions on both hepatic lobules (Fig. 2). To further evaluate these lesions, a colonoscopy was done which revealed (Fig. 3) a prominent ileocecal valve with a mammillated and eroded lesion. The hepatic and intestinal biopsies identified a well-differentiated grade 2 primary NET (Fig. 4) of the ileocecal valve with hepatic metastasis. Treatment was established; he was started on octreotide LAR 30 mg intramuscular monthly and underwent a wide hepatectomy and right hemicolectomy, with initial improvement of the symptoms.

Two years later the follow up visits showed evidence the patient had tumor progression in his liver. This suggests that the octreotide was not sufficient to block the effect of the neuroamines produced by the tumor. Everolimus 10 mg p.o. daily was started in combination with octreotide LAR in an attempt to control symptoms and tumor progression. Also, with the evident progression of hepatic lesions, the patient has so far been taken to four radiofrequency ablations in order to counteract its growth. The Endocrine Service continues to follow this patient and based on current disease evolution, more interventions will most likely be needed.

## Discussion and conclusion

This case shows a differential diagnostic for flushing not usually considered by allergists, CS symptoms are vague and can delay the diagnosis [5, 36]. Given the similar symptoms between NETs and allergic reactions, Pfanzagl et al. created an experimental model of EC cells to better understand these tumors. The model showed that EC cells had an increased response to Ach, which promotes intestinal secretion and motility, in the presence of histamine (HA) acting on H4Rs and H3Rs. This suggests that EC cells might play a role in the intestinal symptoms of diseases related to increased mast cell numbers like food allergies [26]. This shows that the same substances overlap in NETs and allergic reactions. Reason why, it is imperative for doctors to be able to separate benign entities from life-threatening conditions associated with flushing. After all, an early diagnosis helps to initiate an effective treatment to prevent carcinoid heart disease, prevent bowel obstruction, and improve quality of life and mortality.

Flushing of typical CS often involves the face, neck and upper chest [33]. This case had an atypical presentation because the patient had a flush not only in his face, thorax and abdomen but also in the distal part of his upper extremities (Fig. 1c). On the other hand, the duration was longer than expected in midgut tumors, less than one minute [33]. Moreover, he had no other associated symptoms like hepatomegaly or abdominal pain [35], making the diagnosis rather troublesome. This case is also one of the few reports of CS in children associated with high 5-HIAA levels [3, 27, 36]. He had a very advanced disease at the time of diagnosis, in concordance with Navalkele et al., in which less than half of the children and young adults were diagnosed before regional and distant spread of the disease [11].

Finally, it is important to emphasize the fact that SB-NETs are extremely rare in children and because of this, they are seldom considered among the differential diagnosis of allergy-like symptoms, such as flushing. This report will help to expand the limited literature and promote the use of the available tools in the adult world within the context of young children [37]. All this, in order to tackle the lack of standardized care of a rare but treatable disease [7, 11, 12, 36]. After all, an early diagnosis and surgical resection is probably the only curative therapy for NET [21].

## Abbreviations

5-HIAA: 5-hydroxyindoleacetic acid
CS: Carcinoid Syndrome
CT: Computed tomography
NETs: Neuroendocrine tumors
SB-NETs: Small Bowel Neuroendocrine tumors

## References

[1] Allan B, Davis J, Perez E, Lew J, Sola J (2013). Malignant neuroendocrine tumors: incidence and outcomes in pediatric patients. Eur Pediatr Surg.

[2] Strosberg J (2012). Neuroendocrine tumours of the small intestine. Best Pract Res Clin Gastroenterol.

[3] Boston CH, Phan A, Munsell MF, Herzog CE, Huh WW (2015). A comparison between appendiceal and nonappendiceal neuroendocrine tumors in children and young adults: A single-institution experience. J Pediatr Hematol Oncol.

[4] Ladd AP, Grosfeld JL (2006). Gastrointestinal tumors in children and adolescents. Semin Pediatr Surg.

[5] Hassan MM, Phan A, Li D, Dagohoy CG, Leary C, Yao JC (2008). Risk factors associated with neuroendocrine tumors: A US-based case–control study. Int J Cancer.

[6] Howell DL, O’dorisio MS (2012). Management of neuroendocrine tumors in children, adolescents, and young adults. J Pediatr Hematol Oncol.

[7] Khanna G, O’Dorisio SM, Menda Y, Kirby P, Kao S, Sato Y (2008). Gastroenteropancreatic neuroendocrine tumors in children and young adults. Pediatr Radiol.

[8] Cetinkaya RB, Aagnes B, Thiis-Evensen E, Tretli S, Bergestuen DS, Hansen S (2017). Trends in incidence of neuroendocrine neoplasms in Norway: a report of 16,075 cases from 1993 through 2010. Neuroendocrinology.

[9] Dasari A, Shen C, Halperin D, Zhao B, Zhou S, Xu Y, Shih T, Yao JC. Trends in the incidence, prevalence, and survival outcomes in patients with neuroendocrine tumors in the United States. JAMA Oncol. 2017. doi:10.1001/jamaoncol.2017.0589. 10.1001/jamaoncol.2017.0589 PMC5824320 28448665

[10] Hallet J, Law CH, Cukier M, Saskin R, Liu N, Singh S (2015). Exploring the rising incidence of neuroendocrine tumors: a population-based analysis of epidemiology, metastatic presentation, and outcomes. Cancer.

[11] Navalkele P, O’Dorisio MS, O’Dorisio TM, Zamba GK, Lynch CF (2011). Incidence, survival, and prevalence of neuroendocrine tumors versus neuroblastoma in children and young adults: nine standard SEER registries, 1975–2006. Pediatr Blood Cancer.

[12] Diets IJ, Nagtegaal ID, Loeffen J, de Blaauw I, Waanders E, Hoogerbrugge N, Jongmans MCJ (2016). Childhood neuroendocrine tumours: a descriptive study revealing clues for genetic predisposition. Br J Cancer.

[13] Fernández KS, Aldrink JH, Ranalli M, Ruymann FB, Caniano DA (2015). Carcinoid tumors in children and adolescents: risk for second malignancies. J Pediatr Hematol Oncol.

[14] Shenoy S (2014). Primary small-bowel malignancy: update in tumor biology, markers, and management strategies. J Gastrointest Cancer.

[15] Sippel RS, Chen H (2006). Carcinoid Tumours. Surg Oncol Clin N Am.

[16] Yamaguchi T, Manabe N, Tanaka S, Fukumoto A, Shimamoto M, Nakao M, Kamino D, Chayama K (2005). Multiple carcinoid tumours of the ileum preoperatively diagnosed by enteroscopy with the double-balloon technique. Gastrointest Endosc.

[17] Ramachar SM, Hegde N (2015). Small Bowel Carcinoids: A Single Surgeon’s Experience in Southern India. J Clin Diagn Res.

[18] Farley HA, Pommie RF (2016). Surgical Treatment of Small Bowel Neuroendocrine Tumors. Hematol Oncol Clin N Am.

[19] DiSario JA, Burt RW, Vargas H, McWhorter WP (1994). Small bowel cancer: epidemiological and clinical characteristics from a population-based registry. Am J Gastroenterol.

[20] Modlin IM, Sandor A (1997). An analysis of 8305 cases of carcinoid tumours. Cancer.

[21] Yao JC, Hassan M, Phan A, Dagohoy C, Leary C, MaresJE AEK, Fleming JB, Vauthey JN, Rashid A, Evans DB (2008). One hundred years after “carcinoid”: epidemiology of and prognosticf actors for neuroendocrine tumours in 35,825 cases in the United States. J Clin Oncol.

[22] Howe JR, Cardona K, Fraker DL, Kebebew E, Untch BR, Wang YZ, Law CH, Liu EH, Kim MK, Menda Y, Morse BG, Bergsland EK, Strosberg JR, Nakakura EK, Pommier RF (2017). The Surgical Management of Small Bowel Neuroendocrine Tumors: Consensus Guidelines of the North American Neuroendocrine Tumor Society. Pancreas.

[23] de Mestier L, Lardière-Deguelte S, Brixi H, O’Toole D, Ruszniewski P, Cadiot G, Kianmanesh R (2015). Updating the surgical management of peritoneal carcinomatosis in patients with neuroendocrine tumors. Neuroendocrinology.

[24] Negoi I, Paun S, Hostiuc S, Stoica B, Tanase I, Negoi RI, Beuran M (2015). Most small bowel cancers are revealed by a complication. Einstein.

[25] Maglinte DD, O’Connor K, Bessette J, Chernish SM, Kelvin FM (1991). The role of the physician in the late diagnosis of primary malignant tumors of the small intestine. Am J Gastroenterol.

[26] Pfanzagl B, Mechtcheriakova D, Meshcheryakova A, Aberle SW, Pfragner R, Jensen-Jarolim E (2017). Activation of the ileal neuroendocrine tumor cell line P-STS by acetylcholine is amplified by histamine: role of H3R and H4R. Sci Rep.

[27] Dall’Igna P, Ferrari A, Luzzatto C, Bisogno G, Casanova M, Alaggio R (2005). Carcinoid tumor of the appendix in childhood: the experience of two Italian institutions. J Pediatr Gastroenterol Nutr.

[28] Bax ND, Woods HF, Batchelor A, Jennings M (1996). Clinical manifestations of carcinoid disease. World J Surg.

[29] Halperin DM, Shen C, Dasari A, Xu Y, Chu Y, Zhou S, Shih YT, Yao JC (2017). Frequency of carcinoid syndrome at neuroendocrine tumour diagnosis: a population-based study. Lancet Oncol.

[30] Janson ET, Holmberg L, Stridsberg M, Eriksson B, Theodorsson E, Wilander E, Oberg K (1997). Carcinoid tumors: analysis of prognostic factors and survival in 301 patients from a referral center. Ann Oncol.

[31] Niederle MB, Hackl M, Kaserer K, Niederle B (2010). Gastroenteropancreatic neuroendocrine tumours: the current incidence and staging based on the WHO and European Neuroendocrine Tumour Society classification: an analysis based on prospectively collected parameters. Endocr Relat Cancer.

[32] Corpron CA, Black CT, Herzog CE, Sellin RV, Lally KP, Andrassy RJ (1995). A half century of experience with carcinoid tumors in children. Am J Surg.

[33] Hannah-Shmouni F, Stratakis CA, Koch CA (2016). Flushing in (neuro) endocrinology. Rev Endocr Metab Disord.

[34] Clift AK, Faiz O, Goldin R, Martin J, Wasan H, Liedke MO, Schloericke E, Malczewska A, Rindi G, Kidd M, Modlin IM, Frilling A (2017). Predicting the survival of patients with small bowel neuroendocrine tumours: comparison of 3 systems. Endocr Connect.

[35] Spunt SL, Pratt CB, Rao BN, Pritchard M, Jenkins JJ, Hill DA (2000). Childhood carcinoid tumors: the St Jude Children’s Research Hospital experience. J Pediatr Surg.

[36] Boudreaux JP, Klimstra DS, Hassan MM, Woltering EA, Jensen RT, Goldsmith SJ (2010). The NANETS consensus guideline for the diagnosis and management of neuroendocrine tumors: well-differentiated neuroendocrine tumors of the jejunum, ileum, appendix, and cecum. Pancreas.

[37] Johnson PR (2014). Gastroenteropancreatic neuroendocrine (carcinoid) tumors in children. Semin Pediatr Surg.

